# Supplementary material for: Evolution of intrinsically disordered regions in vertebrate galectins for phase separation
Source: EMBO Rep. 2026 Feb 2;27(5):1254–69. doi: 10.1038/s44319-026-00692-w (PMC12979664; doi:10.1038/s44319-026-00692-w)
Supplement: Supplementary file 1 — Appendix [file 44319_2026_692_MOESM1_ESM.pdf]

Appendix

for

Evolution of intrinsically disordered regions in vertebrate galectins for phase separation

Yu-Hao Lin, Yu-Chen Chen, Yung-Chen Sun, and Jie-rong Huang*

*Correspondence author: Jie-rong Huang

Email: jierongh@nycu.edu.tw

Table of contents

Appendix Methods……………………………………….……....3
Appendix Figure S1……..…………………………………………..4
Appendix Figure S2…………..……………………………………..5
Appendix Figure S3………………..………………………………..6
Appendix Figure S4……………………..…………………………..7
Appendix Figure S5…………………………….………………..8
Appendix Figure S6………………………………………..………..9
Appendix Figure S7…………………………………………………..10
Appendix Figure S8…………………………………………………..11

Appendix Methods

Protein expression and purification

Fusion proteins (galectin variants and His_6-SUMO tags) were purified using nickel-charged immobilized metal-ion affinity chromatography (IMAC) columns (Qiagen). Imidazole was removed using a PD-10 column (Cytiva). Subsequently, His_6-Ulp1$^{403–621}$ protease was added to the protein solution at a concentration of 5 µg/ml for 1 h at 4 °C to cleave the His_6-SUMO tag from the galectin-3 variants. After enzyme digestion, the solution was injected into a nickel-charged IMAC column once more to separate the cleaved components and the flow-through containing the protein of interest was collected. The collected protein was loaded onto a HiLoad Superdex 75 pg gel-filtration column (Cytiva) with an FPLC system or a PD-10 column to exchange the appropriate buffer. Samples were flash-frozen in liquid nitrogen and stored at −80 °C before use.

NMR data collection and analysis

^{1}H-^{15}N HSQC spectra were recorded using the standard pulse sequence with WATERGATE solvent suppression (1, 2). The ^{15}N dimension was sampled with 128 increments and zero-filled to 1024 points in NMRPipe prior to Fourier transformation; the ^{1}H dimension was acquired with 2048 points. Chemical shifts were assigned using standard HNCA, HN(CO)CA, HNCO, HN(CA)CO, CBCA(CO)NH, and HNCACB experiments acquired with non-uniform sampling (25%) (3, 4). Spin relaxation rate experiments were performed using standard pulse sequences (1, 2, 5). ^{15}N R_2 and R_1 rate constants were determined using delay times of 17, 34, 51, 68, 85, and 102 ms, and 100, 200, 300, 600, 800, and 1000 ms, respectively. Peak intensities were fitted to an exponential decay relative to the delay times with a Monte Carlo procedure to estimate fitting errors. All dynamics data were collected in an interleaved manner with an interscan delay of 3 s. All NMR data were recorded on a Bruker AVIII 850 MHz spectrometer with a cryogenic probe at 303 K unless otherwise stated.

Data were processed using NMRPipe (6) and analyzed with SPARKY (7). Peak intensities and errors were determined using the non-linear line-shape analysis (nlinLS) function in NMRPipe based on the noise of the spectra. Intensity ratios were normalized to the number of scans. The average chemical shift difference ($\Delta\delta^{HN}$) was calculated using

$$\Delta\delta^{\mathrm{HN}} = \sqrt{\frac{(\Delta\delta_{\mathrm{H}})^2 + \left(\frac{1}{5}\Delta\delta_{\mathrm{N}}\right)^2}{2}}$$

where $\Delta\delta_H$ and $\Delta\delta_N$ are the differences in chemical shift between two HSQC spectra for the amide proton and nitrogen, respectively.

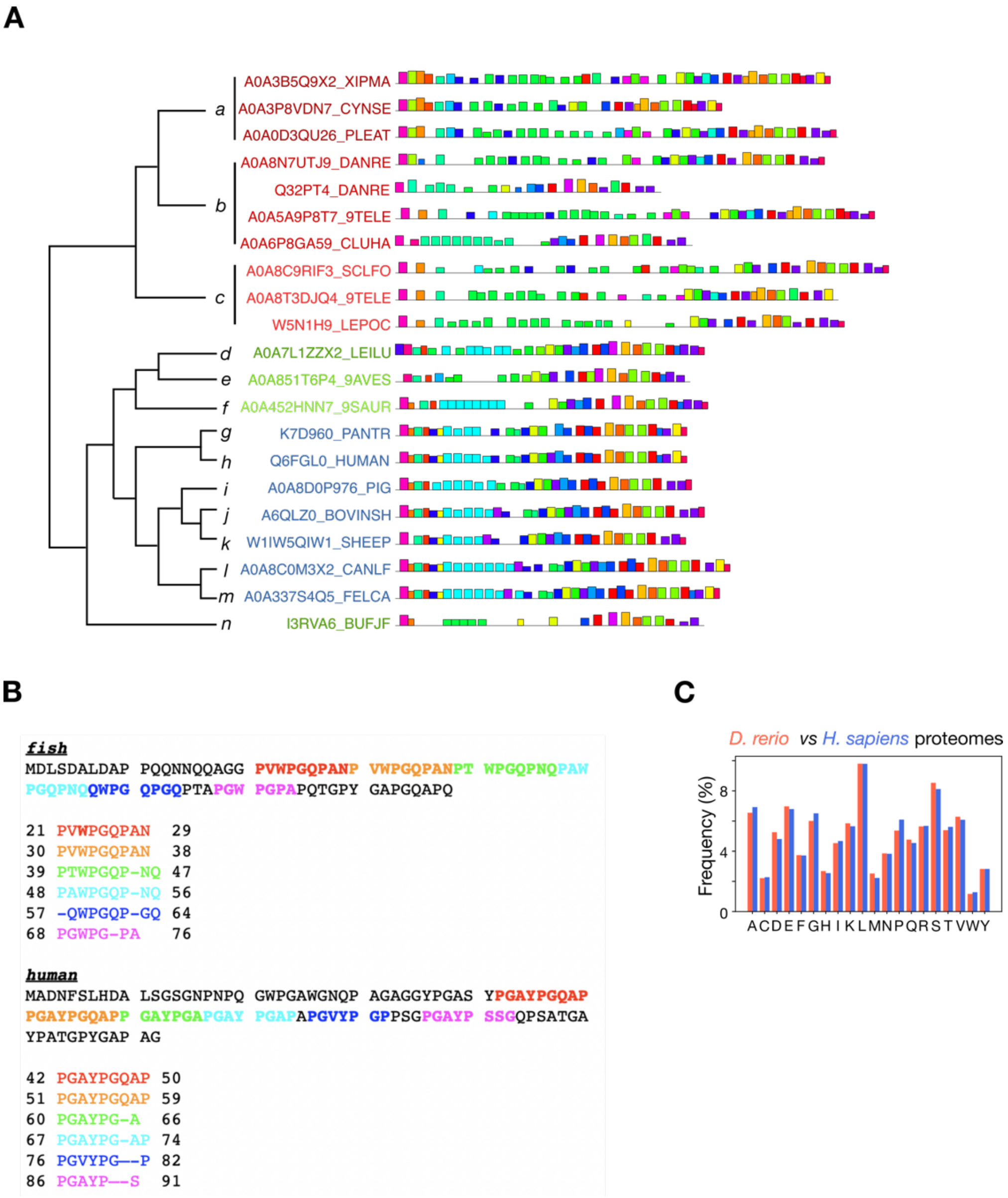

Appendix Figure S1. Sequence motifs of representative vertebrates and amino acid sequences of the studied zebrafish and human galectins. (A) Phylogenetic relationships and MEME motifs of selected galectin homologs across vertebrate clades. Branches labeled (*a*), (*b*), and (*c*) represent teleost fishes from *Euteleosteomorpha*, *Otomorpha* (within *Clupeocephala*), and *Osteoglossocephala*, respectively. Avian lineages are illustrated by (*d*) *Neognathae* and (*e*) *Paleognathae*, while (*f*) denotes a representative from *Sauria* (reptiles). Mammalian and amphibian sequences include chimpanzee (*g*), human (*h*), pig (*i*), bovine (*j*), sheep (*k*), dog (*l*), cat (*m*), and frog (*n*). Each sequence is indicated by its UniProt ID, and the colored blocks to the right represent distinct motifs identified with the MEME suite. The tree (left) is shown in simplified form to highlight phylogenetic relationships among selected taxa. (B) Sequences of zebrafish (UniProt ID: Q6TGN4) and human (P17931) IDR-tethered galectins; the repeated motifs in the intrinsically disordered regions are highlighted and aligned. (C) Amino-acid composition of *Danio rerio* and *Homo sapiens* proteomes.

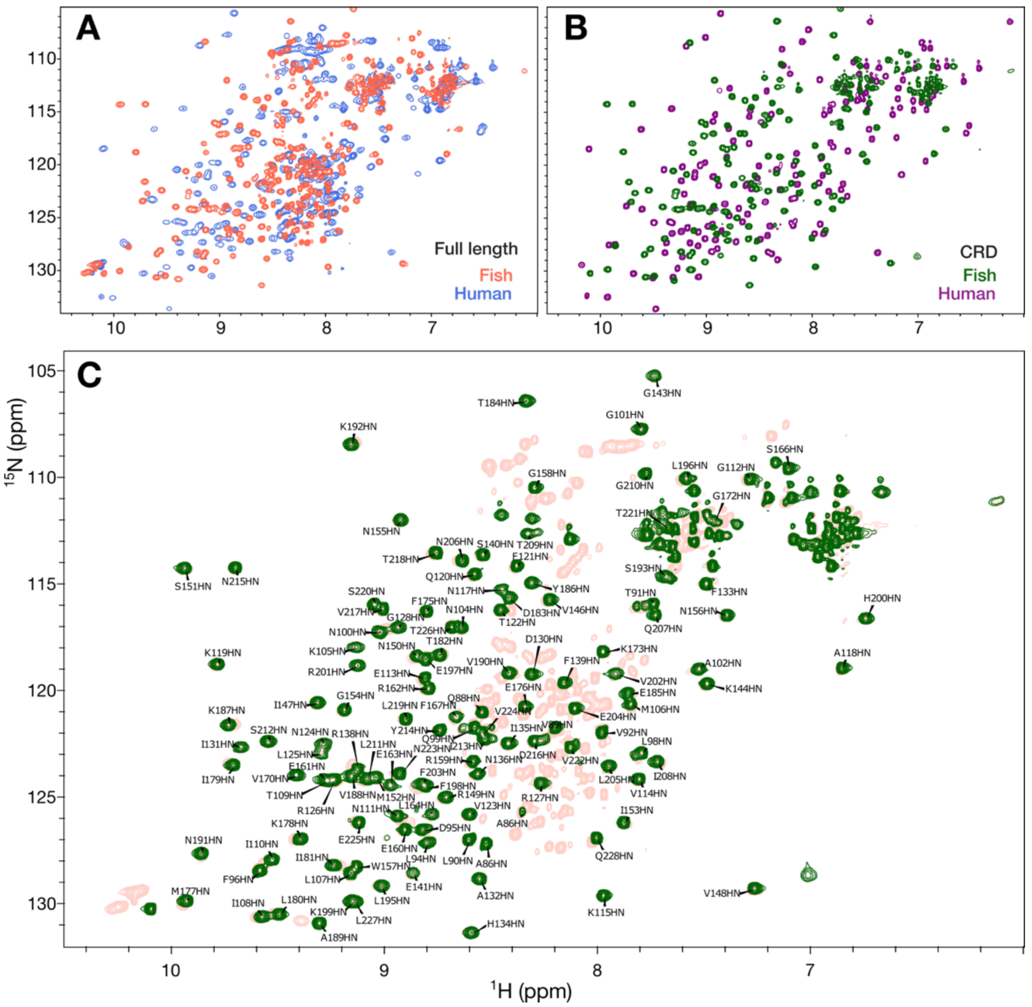

Appendix Figure S2. ^{1}H-^{15}N HSQC spectra of zebrafish and human IDR-tethered galectins. Overlaid ^{1}H-^{15}N HSQC spectra of (A) full-length and (B) carbohydrate-recognition domain (CRD)-only constructs of human and zebrafish IDR-tethered galectins. (C) Chemical shift assignments of the CRD of zebrafish IDR-tethered galectin (deposited in the BMRB (52443)) overlaid on the spectrum of the full-length protein (light red).

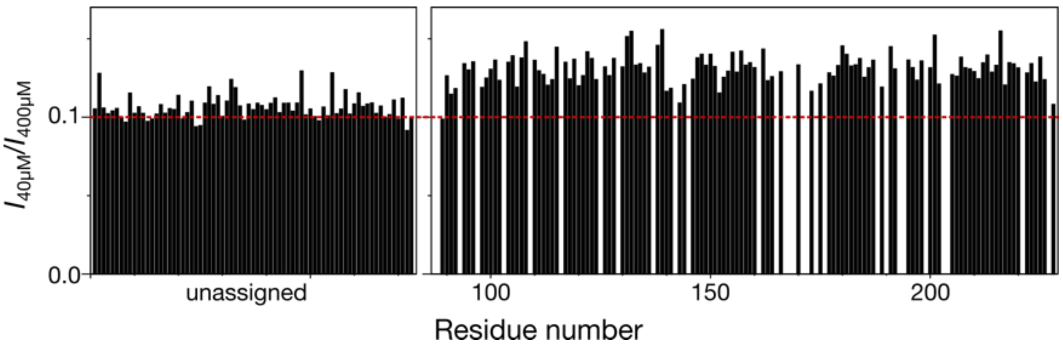

Appendix Figure S3. NMR peak intensity ratios between 40 µM and 400 µM zebrafish galectin samples. The red line shows the signal intensity ratio expected from the molar ratio (i.e., ignoring any intermolecular interactions).

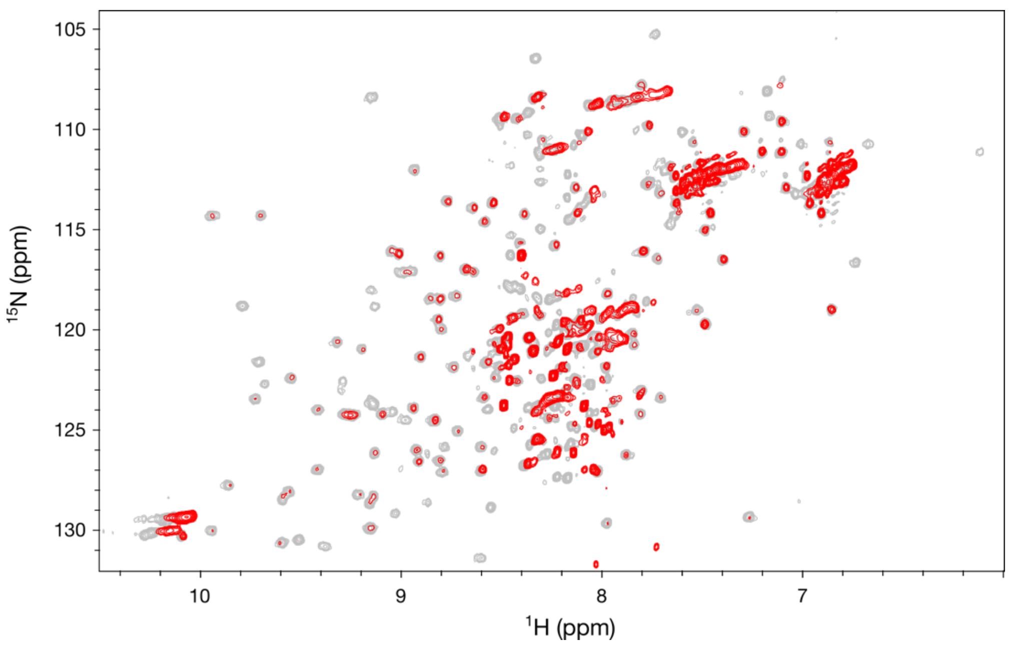

Appendix Figure S4. Overlaid ^{1}H-^{15}N HSQC spectrum of the IDR-augmented zebrafish galectin construct (zfGal^{aug}, red) and wild-type zfGal (gray), both at 40 µM, shown on the same intensity scale (same contour levels).

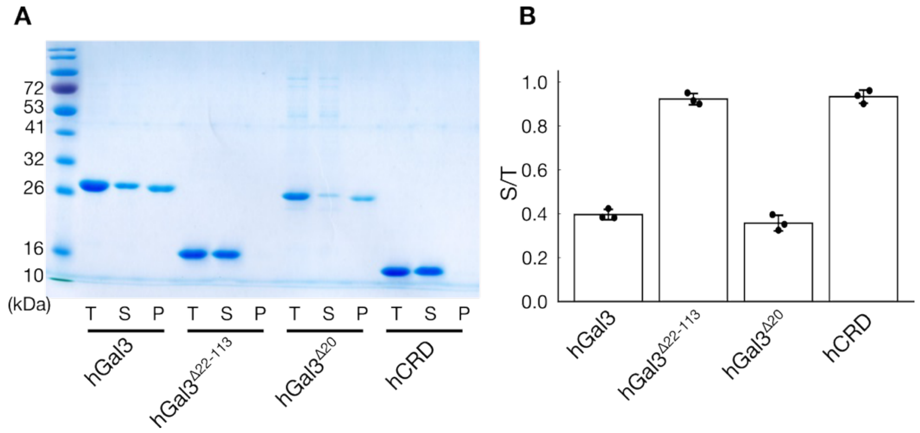

Appendix Figure S5. LPS-induced agglutination depends on the conserved galectin-3 N-terminus. Wild-type human galectin-3 (hGal3) is compared with two N-terminal variants: hGal3$^{\Delta 22\text{–}113}$, in which N-terminal domain (NTD) residues 22–113 are deleted, leaving only residues 1–21 N-terminal to the CRD, and hGal3$^{\Delta 20}$, in which NTD residues 1–20 are deleted, as well as the isolated carbohydrate-recognition domain (hCRD). (A) Coomassie-stained SDS–PAGE of the total reaction (T) and the corresponding supernatant (S) and pellet (P) fractions after incubation with LPS and centrifugation (conditions in Methods). (B) Protein in S and T quantified by Bradford assay (n = 3; mean ± s.d.).

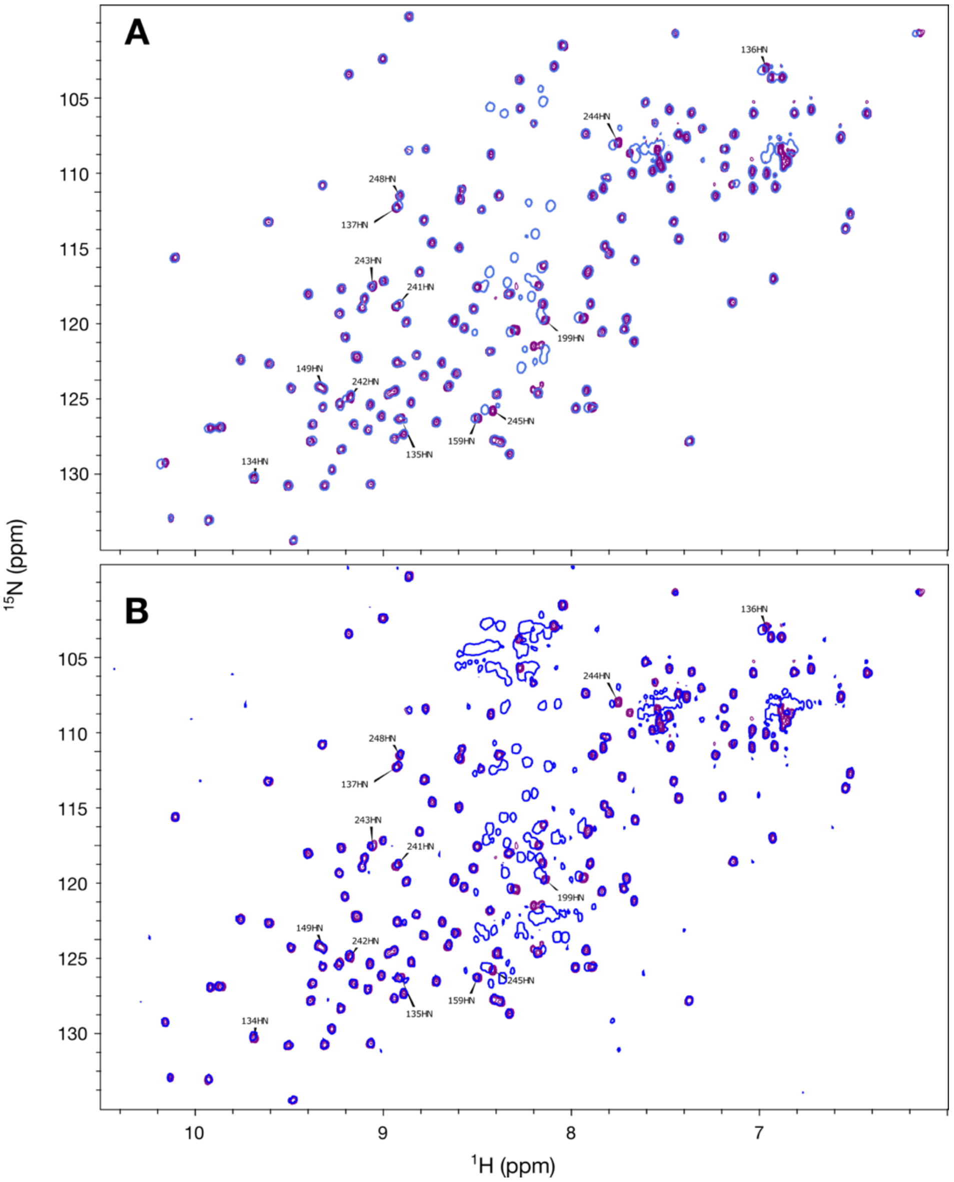

Appendix Figure S6. ^{1}H-^{15}N HSQC spectra of human galectin-3 variants (A) without residues 22–113 (hGal3$^{\Delta 21\text{-}113}$, blue contours) and (B) with all tryptophans and tyrosines in the intrinsically disordered region replaced by glycine (hGal3$^{WY/G}$, blue contours) overlaid onto the spectrum of the human CRD-only construct (purple). The peaks that shift the most between constructs are labeled (except those adjacent between the CRD and N-terminal IDRs).

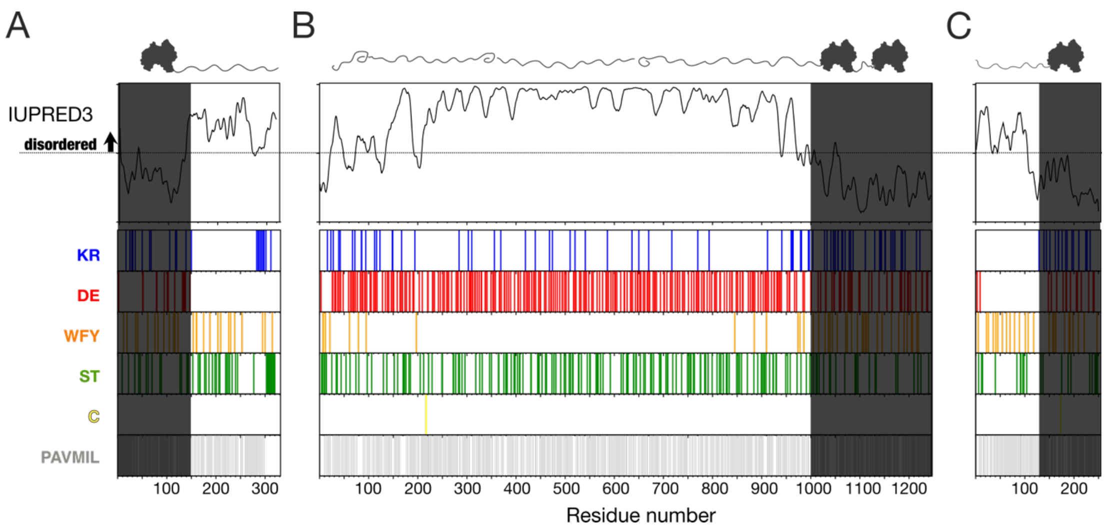

Appendix Figure S7. Comparison of carbohydrate recognition domains (CRDs) tethered to intrinsically disordered regions (IDRs) across three evolutionarily distant species. (A) A galectin-like protein from the plant *Rhodamnia argentea* (UniProt: A0A8B8QVV1), featuring an IDR appended to the C terminus with only positive charges. (B) A tandem-repeat galectin from *Caenorhabditis elegans* (UniProt: Q20684) with a long negative-charge dominant IDR. (C) Human galectin-3 (UniProt: P17931) is also shown for reference; its IDR contains only two negatively charged residues. Intrinsic disorder was predicted using IUPRED3. Colors indicate residues with different physicochemical properties (e.g., positively or negatively charged, aromatic).

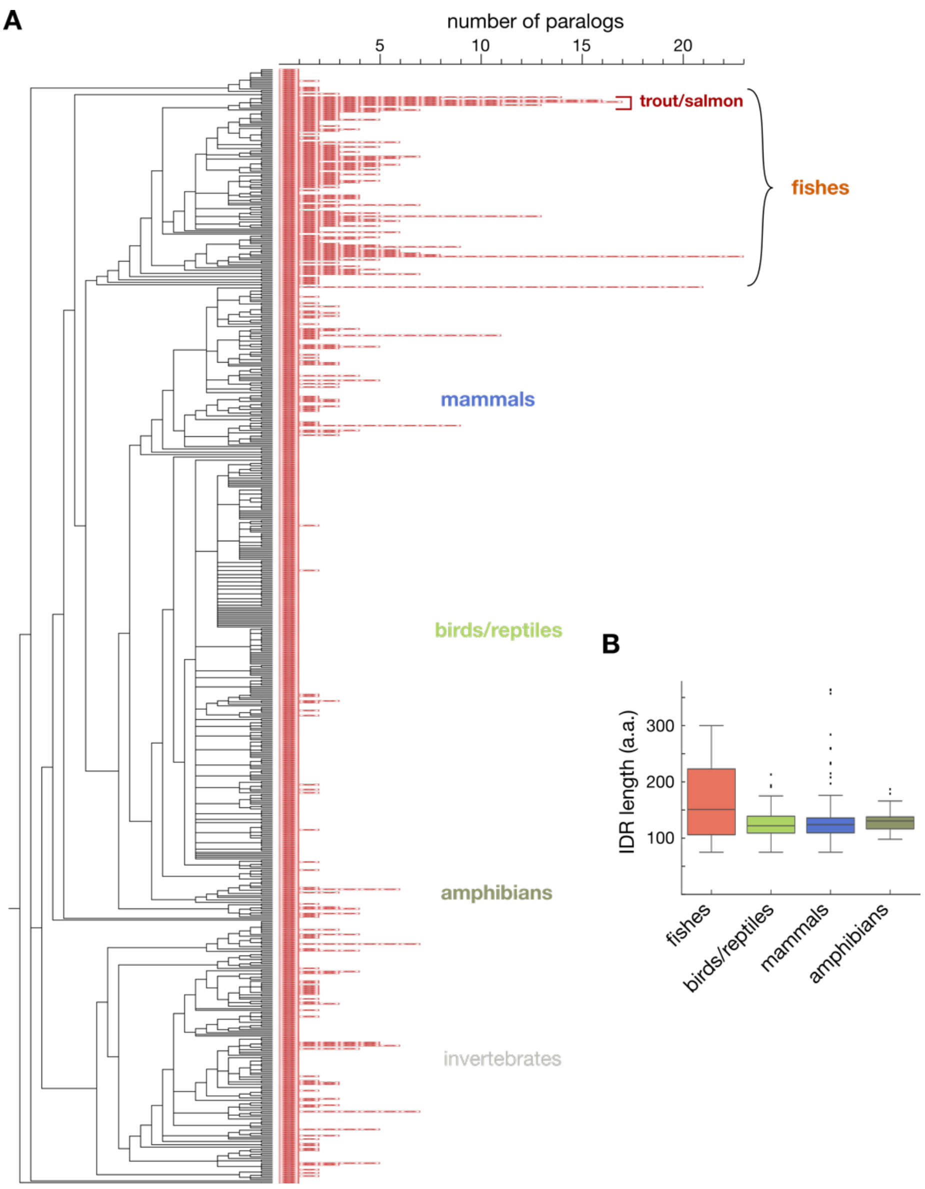

Appendix Figure S8. (A) Number of IDR-tethered galectin paralogs (red blocks) in different species. The phylogenetic tree is the same as shown in Dataset EV2. (B) The boxplot lengths distribution of different vertebrates.

Reference

1. M. Piotto, V. Saudek, V. Sklenar, Gradient-tailored excitation for single-quantum NMR spectroscopy of aqueous solutions. *J Biomol NMR* **2**, 661-665 (1992).
2. G. Bodenhausen, D. J. Ruben, Natural abundance N-15 NMR by enhanced heteronuclear spectroscopy. . *Chem. Phys. Letters* **69**, 185-189 (1980).
3. S. G. Hyberts, A. G. Milbradt, A. B. Wagner, H. Arthanari, G. Wagner, Application of iterative soft thresholding for fast reconstruction of NMR data non-uniformly sampled with multidimensional Poisson Gap scheduling. *J Biomol NMR* **52**, 315-327 (2012).
4. S. G. Hyberts, D. P. Frueh, H. Arthanari, G. Wagner, FM reconstruction of non-uniformly sampled protein NMR data at higher dimensions and optimization by distillation. *J Biomol NMR* **45**, 283-294 (2009).
5. N. A. Farrow *et al.*, Backbone dynamics of a free and phosphopeptide-complexed Src homology 2 domain studied by 15N NMR relaxation. *Biochemistry* **33**, 5984-6003 (1994).
6. F. Delaglio *et al.*, NMRPipe: a multidimensional spectral processing system based on UNIX pipes. *J Biomol NMR* **6**, 277-293 (1995).
7. T. D. Goddard, D. G. Kneller (2005) Sparky 3. San Francisco, University of California.
