# Supplementary material for: Evolution of intrinsically disordered regions in vertebrate galectins for phase separation
Source: EMBO Rep. 2026 Feb 2;27(5):1254–69. doi: 10.1038/s44319-026-00692-w (PMC12979664; doi:10.1038/s44319-026-00692-w)
Supplement: Supplementary file 3 — Dataset EV1 [file 44319_2026_692_MOESM3_ESM.zip › DatasetEV1/Dataset EV1.docx]

**Dataset EV1.** Phylogenetic relationships and sequence characteristics of galectin-3 proteins. This phylogenetic tree represents the evolutionary relationships among galectin-3 proteins as sourced from the Orthologous Matrix (OMA) database. The pie charts at each node depict the amino-acid composition of disordered sequences. The level of structural disorder is accessed using IUPRED3.
