# Supplementary figures and images for: Evolution of intrinsically disordered regions in vertebrate galectins for phase separation
Source: EMBO Rep. 2026 Feb 2;27(5):1254–69. doi: 10.1038/s44319-026-00692-w (PMC12979664; doi:10.1038/s44319-026-00692-w)

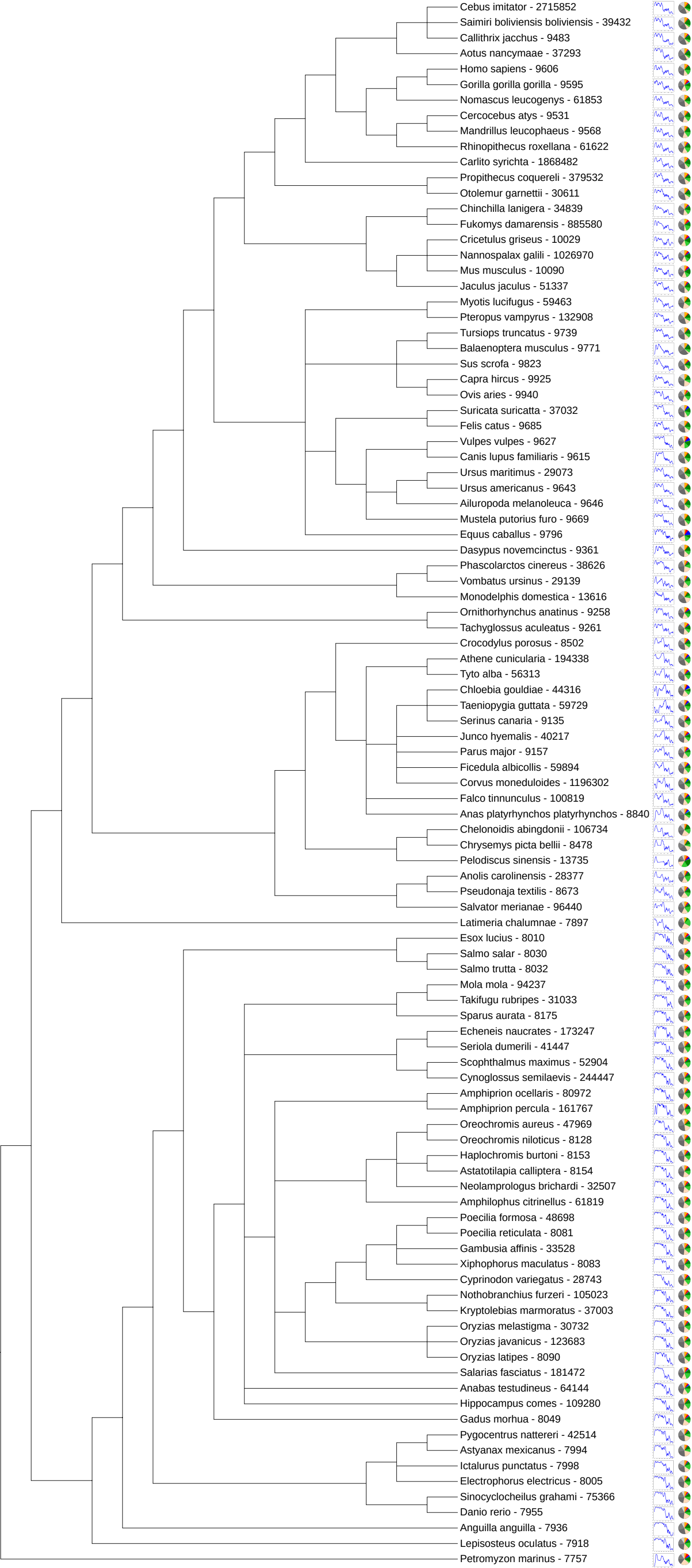

Supplement: Supplementary file 3 — Dataset EV1 [file 44319_2026_692_MOESM3_ESM.zip › DatasetEV1/ED_1_OMA_gal3_tree_itol_pie_iupred.pdf]

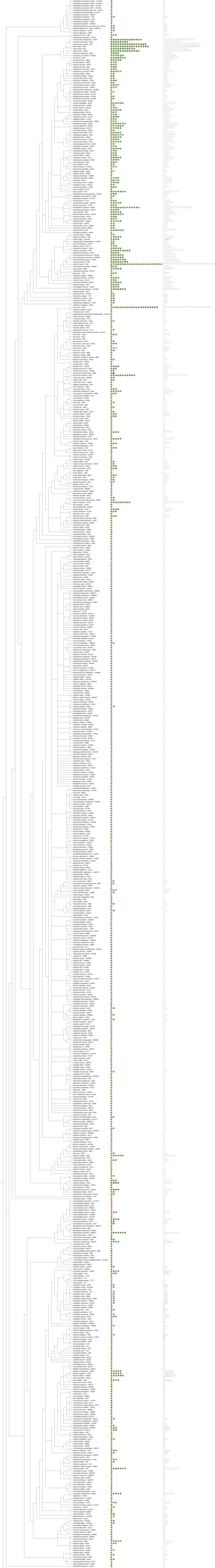

Supplement: Supplementary file 4 — Dataset EV2 [file 44319_2026_692_MOESM4_ESM.zip › DatasetEV2/ED_2_1321_tree.pdf]

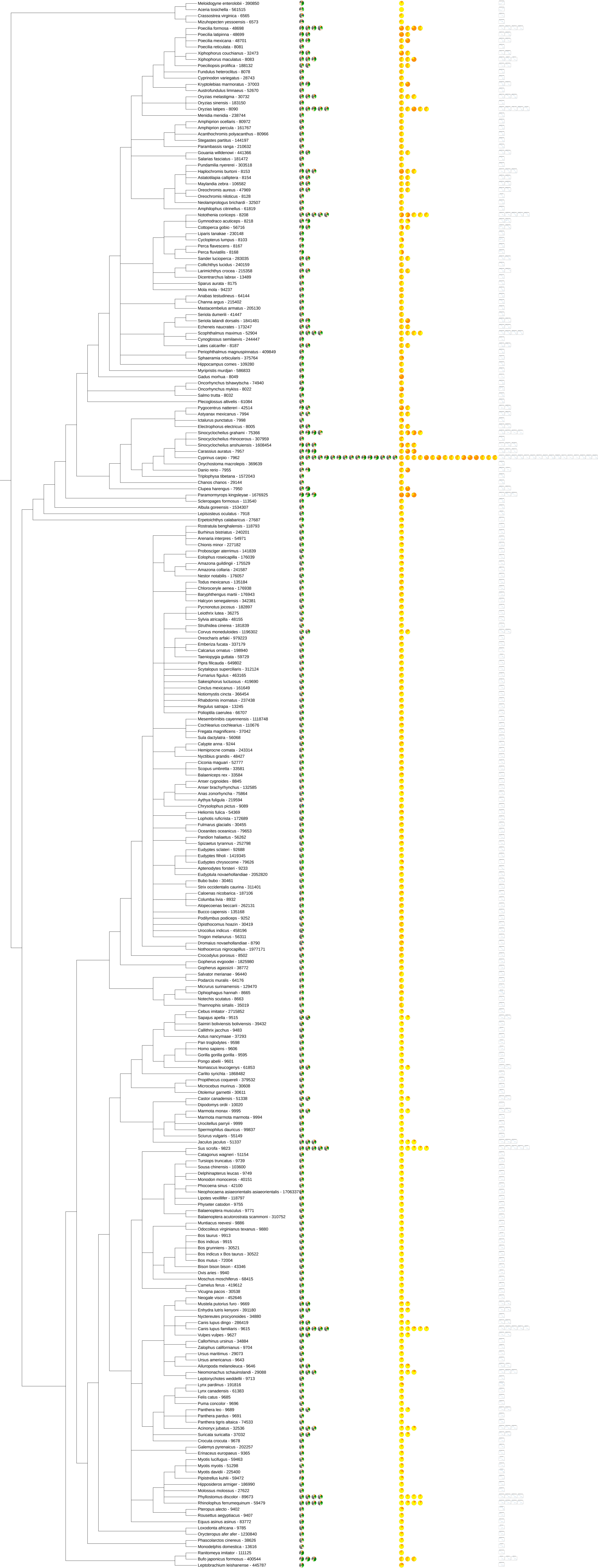

Supplement: Supplementary file 5 — Dataset EV3 [file 44319_2026_692_MOESM5_ESM.zip › DatasetEV3/ED_3_wyf_delargerkr_tree.pdf]

## Slide 1
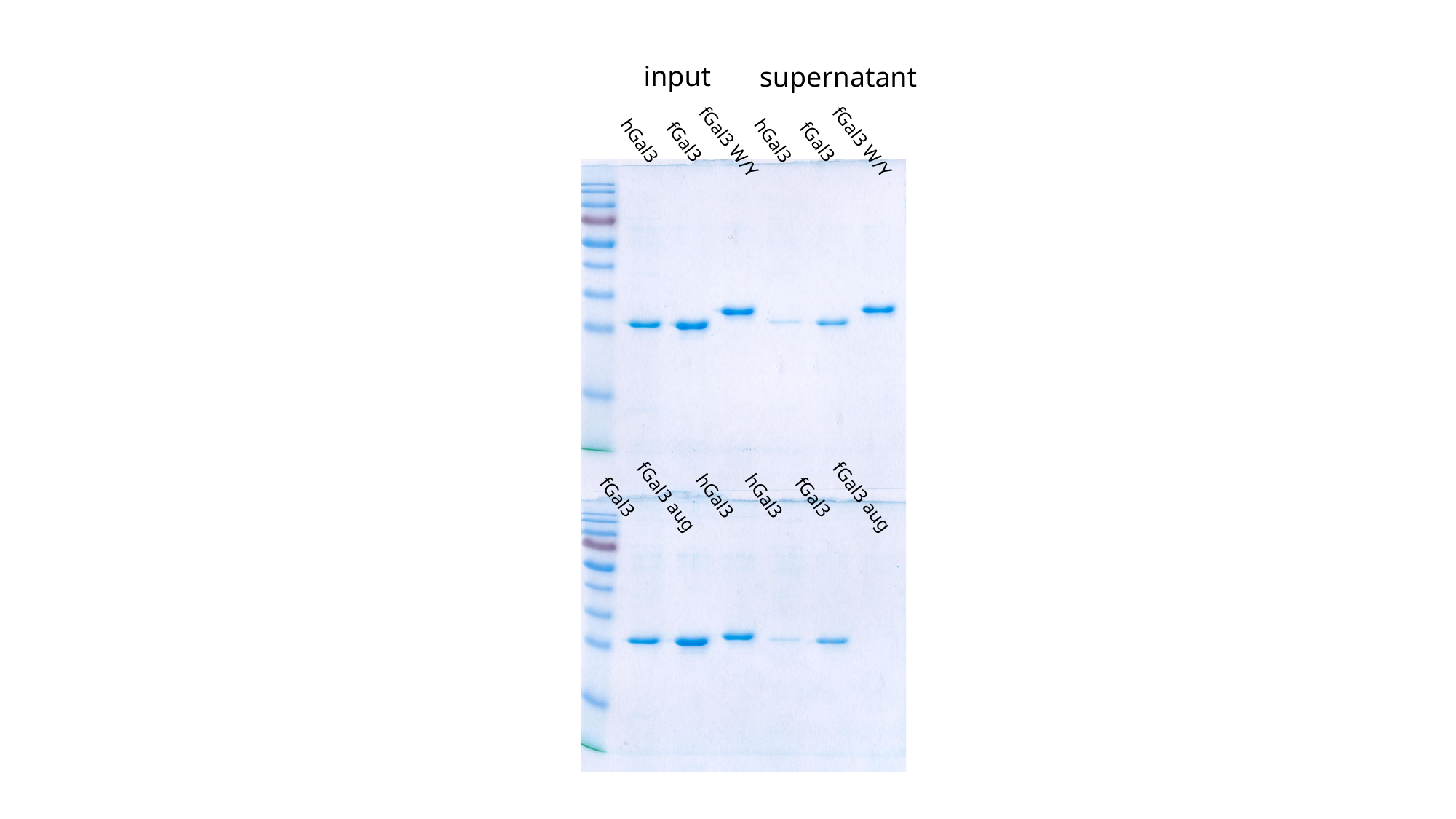

input
supernatant
hGal3
hGal3
fGal3
fGal3 W/Y
fGal3
fGal3 W/Y
hGal3
hGal3
fGal3
fGal3 aug
fGal3
fGal3 aug

Supplement: Supplementary file 10 — Source data Fig. 4 [file 44319_2026_692_MOESM10_ESM.zip › FIG_4/4G/SDS.pptx]
