# Supplementary material for: Evolution of intrinsically disordered regions in vertebrate galectins for phase separation
Source: EMBO Rep. 2026 Feb 2;27(5):1254–69. doi: 10.1038/s44319-026-00692-w (PMC12979664; doi:10.1038/s44319-026-00692-w)
Supplement: Supplementary file 4 — Dataset EV2 [file 44319_2026_692_MOESM4_ESM.zip › DatasetEV2/Dataset EV2.docx]

**Dataset EV2.** Phylogenetic relationships and sequence composition of IDR-tethered galectins. The phylogenetic relationships of the intrinsically disordered region (IDR)-tethered galectins among various species, based on selection criteria outlined in Fig. 1A. The pie charts at each node represent the amino acid composition of these disordered sequences. The analysis of the structural disorder level was performed using the IUPRED3.
