# Supplementary material for: Evolution of intrinsically disordered regions in vertebrate galectins for phase separation
Source: EMBO Rep. 2026 Feb 2;27(5):1254–69. doi: 10.1038/s44319-026-00692-w (PMC12979664; doi:10.1038/s44319-026-00692-w)
Supplement: Supplementary file 5 — Dataset EV3 [file 44319_2026_692_MOESM5_ESM.zip › DatasetEV3/Dataset EV3.docx]

**Dataset EV3.** Phylogenetic relationships and sequence composition of IDR-tethered galectins with criteria based on human galectin-3 sequence. The phylogenetic relationships of intrinsically disordered region (IDR)-tethered galectins among various species were selected based on the criteria outlined in Fig. 1A. The pie charts at each node represent the amino acid composition of these disordered sequences. The composition of aromatic residues is also analyzed. Color-coded based on Fig. 1B. The analysis of the structural disorder level was performed using the IUPRED3.
