# Supplementary material for: Evolution of intrinsically disordered regions in vertebrate galectins for phase separation
Source: EMBO Rep. 2026 Feb 2;27(5):1254–69. doi: 10.1038/s44319-026-00692-w (PMC12979664; doi:10.1038/s44319-026-00692-w)
Supplement: Supplementary file 6 — Dataset EV4 [file 44319_2026_692_MOESM6_ESM.zip › DatasetEV4/Dataset EV4.docx]

**Dataset EV4.** Interactive analysis of repeated sequence motifs in IDR-tethered galectins. This interactive HTML file presents a comprehensive sequence motif analysis of IDR-tethered galectins, using the MEME Suite (version 5.5.2). The analysis identifies repeated protein motifs. Results include detailed motif statistics, sequence alignments, and visualization options, aiding in the understanding of motif prevalence and distribution across the studied species.
