# Supplementary material for: Evolution of intrinsically disordered regions in vertebrate galectins for phase separation
Source: EMBO Rep. 2026 Feb 2;27(5):1254–69. doi: 10.1038/s44319-026-00692-w (PMC12979664; doi:10.1038/s44319-026-00692-w)
Supplement: Supplementary file 11 — Source data Fig. 5 [file 44319_2026_692_MOESM11_ESM.zip › FIG_5/5C/photos_Aug_wLPS_wLac.pptx]

## Slide 1
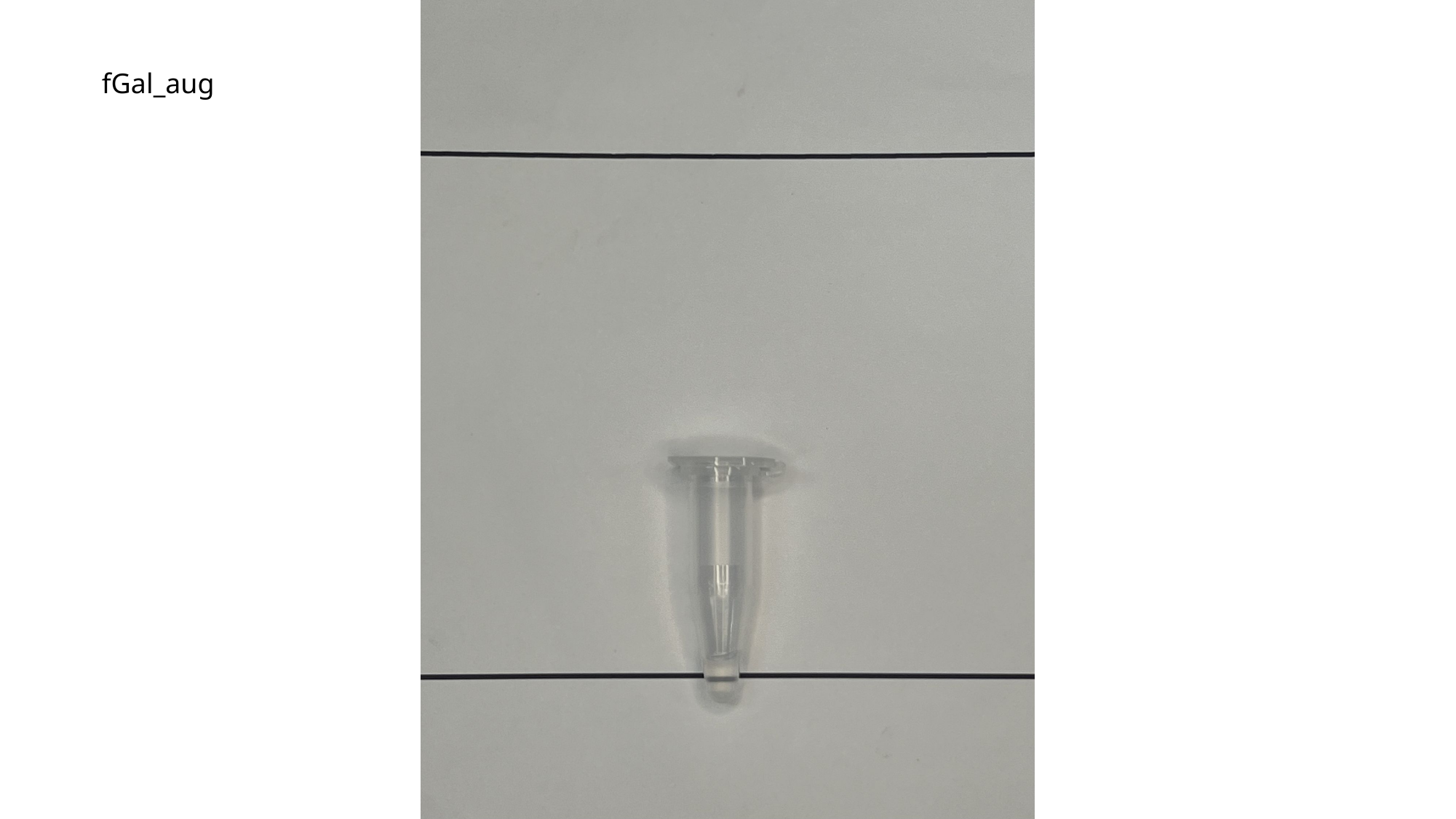

fGal_aug

## Slide 2
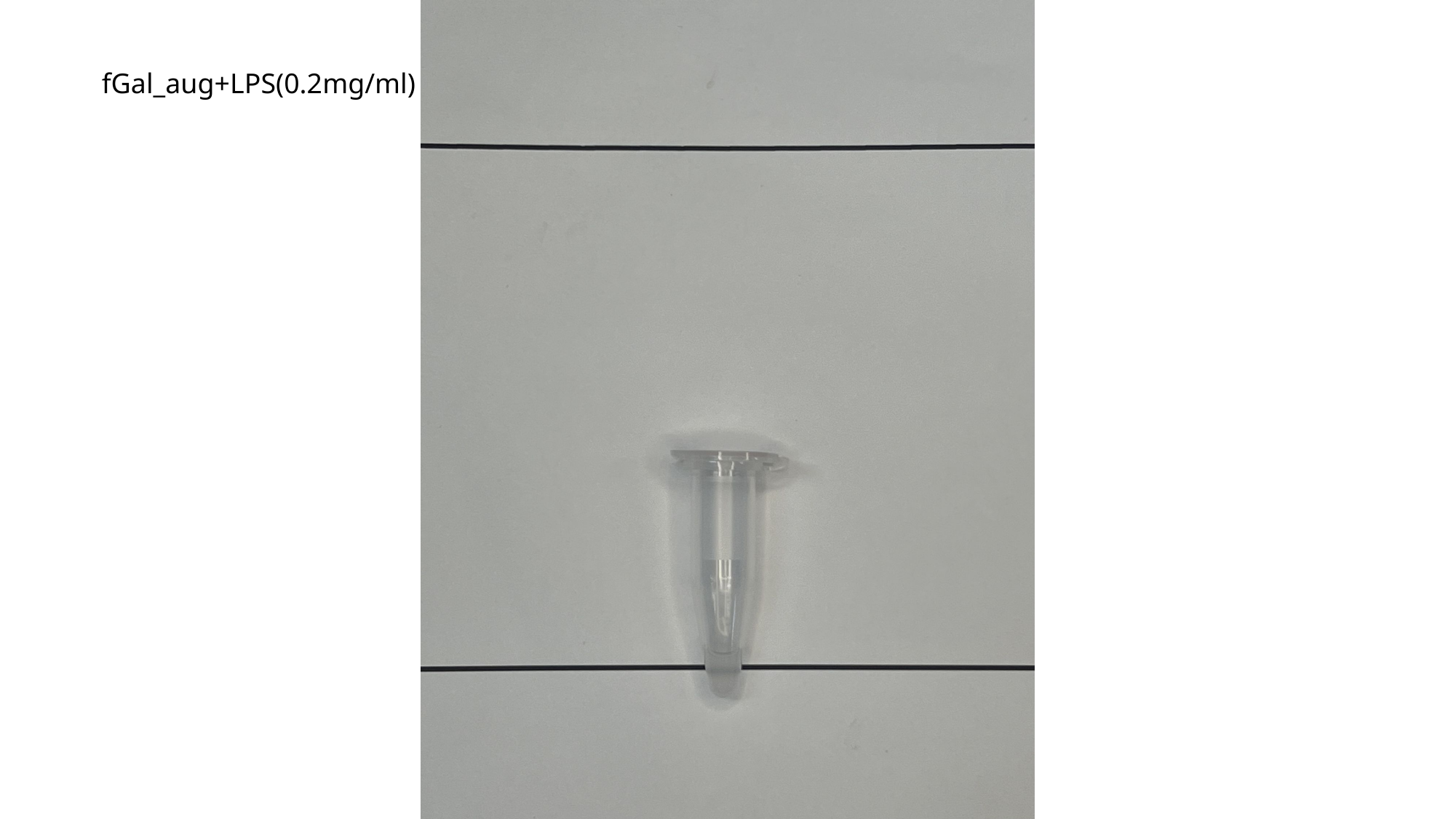

fGal_aug+LPS(0.2mg/ml)

## Slide 3
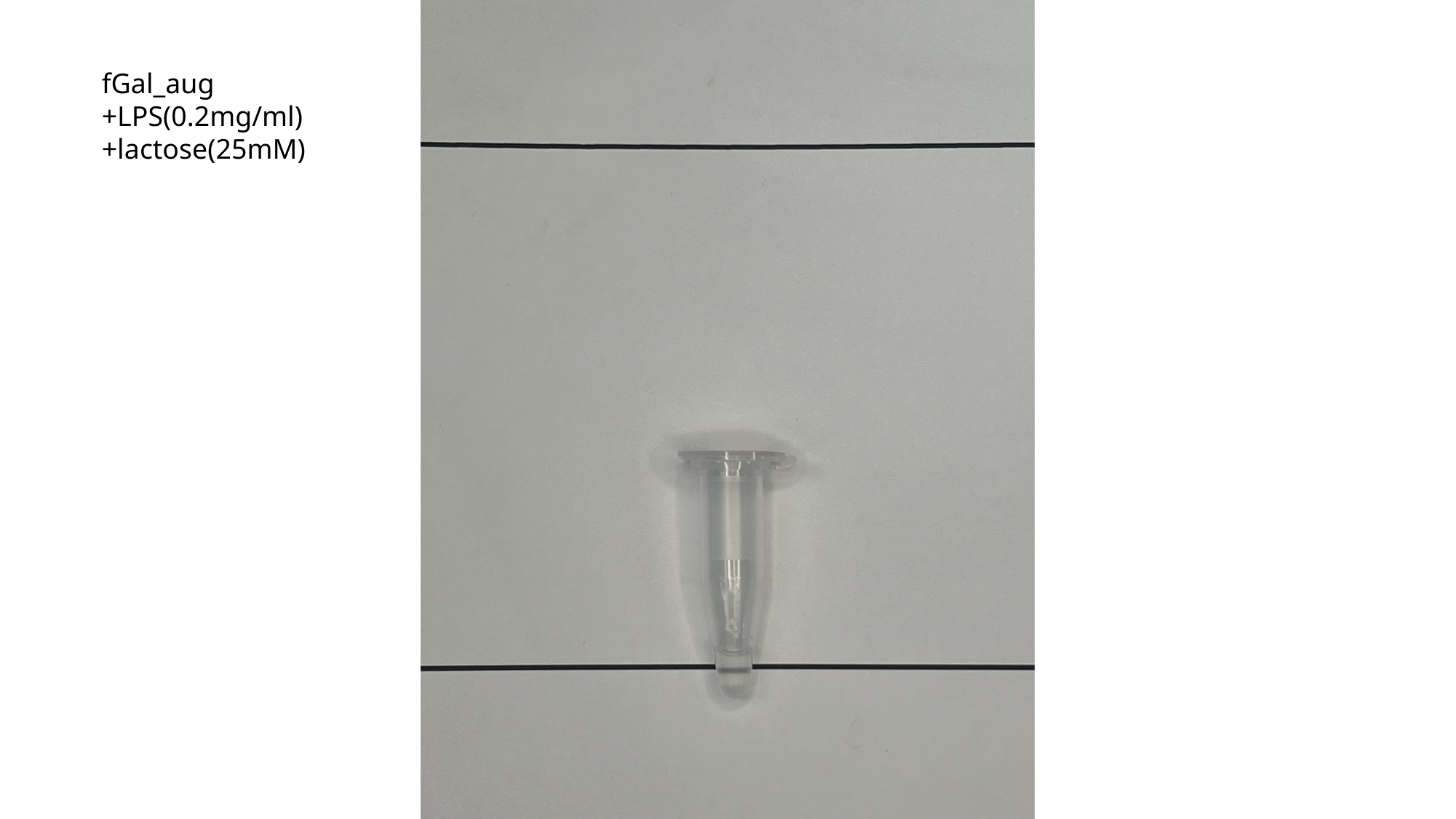

fGal_aug
+LPS(0.2mg/ml)
+lactose(25mM)

## Slide 4
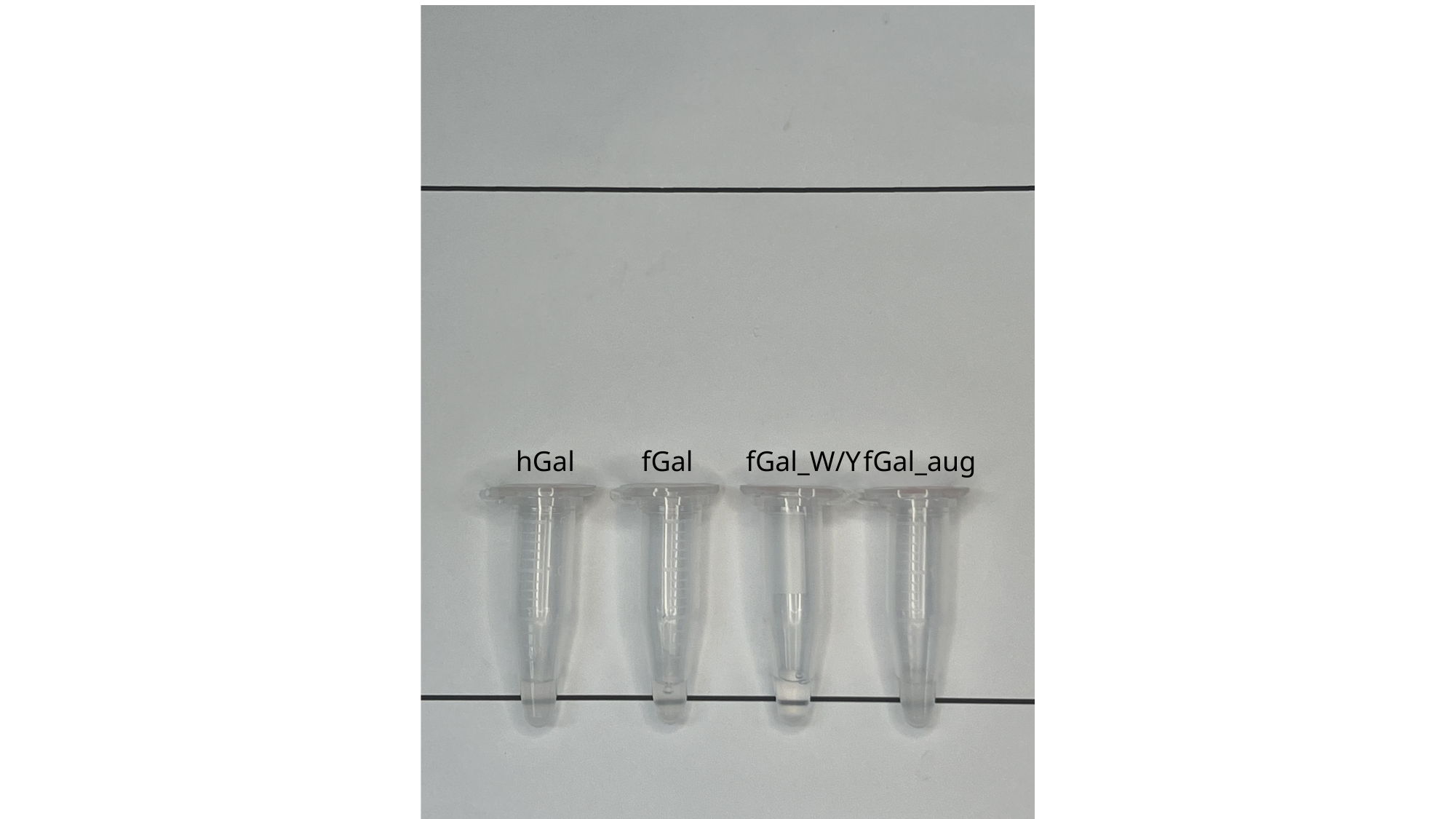

hGal
fGal
fGal_W/Y
fGal_aug

## Slide 5
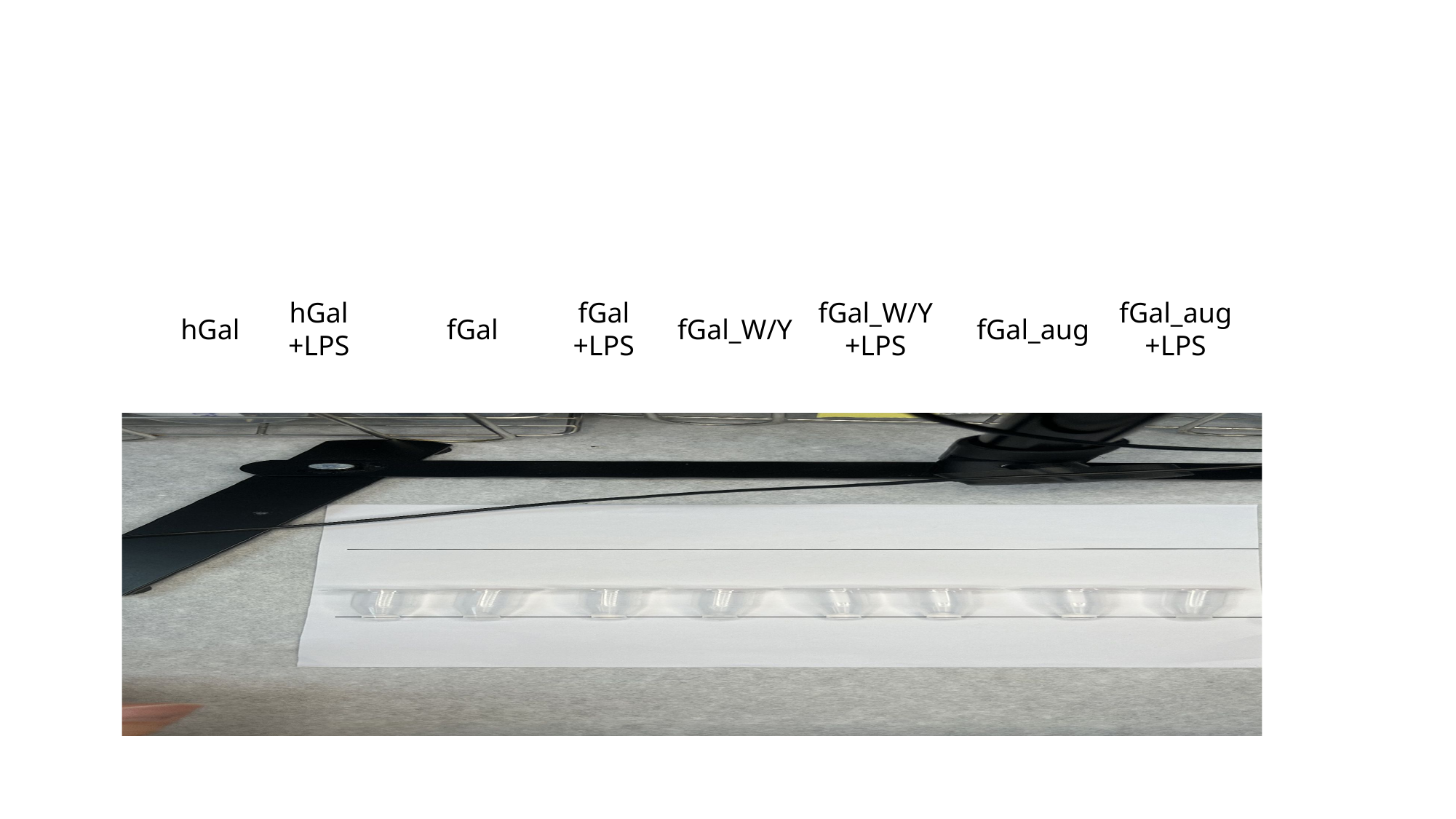

hGal
+LPS
fGal
+LPS
fGal_W/Y
+LPS
fGal_aug
+LPS
hGal
fGal
fGal_W/Y
fGal_aug
