# Supplementary material for: Evolution of intrinsically disordered regions in vertebrate galectins for phase separation
Source: EMBO Rep. 2026 Feb 2;27(5):1254–69. doi: 10.1038/s44319-026-00692-w (PMC12979664; doi:10.1038/s44319-026-00692-w)
Supplement: Supplementary file 13 — Expanded View Figures [file 44319_2026_692_MOESM13_ESM.pdf]

Expanded View Figures

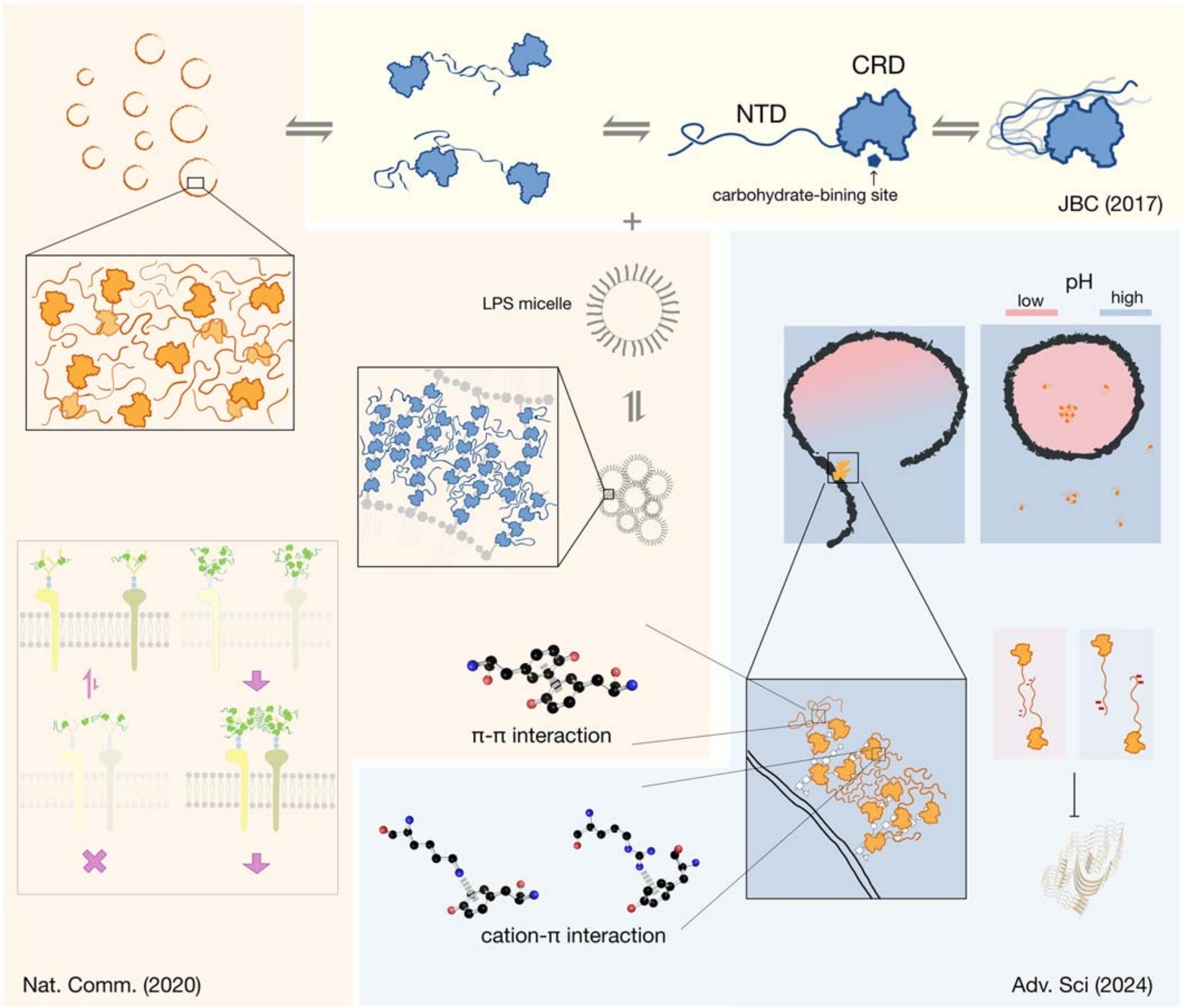

Figure EV1. Summary of previous studies on galectin-3 self-association.

Background shading groups panels by publication: yellow: JBC (Lin et al, 2017); peach: Nat. Commun. (Chiu et al, 2020); blue: Adv. Sci. (Sun et al, 2024). Galectin-3's intrinsically disordered N-terminal domain (NTD) forms transient intra- and intermolecular contacts with the non-carbohydrate-binding face of its carbohydrate-recognition domain (CRD; the NTD-binding face) and also self-associates (yellow). These interactions drive phase separation (PS) and enable galectin-3-mediated agglutination, assessed with lipopolysaccharide (LPS) micelles. Agglutination is supported by π-π interactions among aromatic residues within the NTD (peach) and by cation-π interactions between these aromatics and conserved, positively charged residues on the NTD-binding face. Two conserved acidic residues in the NTD modulate self-association in a pH-dependent manner (blue).

 © The Author(s)
